# Transfusion of Lamb’s Blood—Results

**Published:** 1875-01

**Authors:** 


Transfusion of Lamb's Blood in the Human Subject.— * * The above statement shows that we have operated five times for phthisis, three times for chronic anæmia, and once for hemorrhage, with this result: that the operation was a failure in six cases (1, 2, 3, 4, 5, 9); it caused the death of one patient (7), and two patients only (6 and 8) were permanently benefited. Our experiments, therefore, do not corroborate Dr. Hasse's observations, although some of our patients offered the most favorable conditions for a good result (cases of simple anæmia). All the patients experienced an improvement of their feeling and appetite, for the four to six weeks following the transfusion. This temporary benefit, together with the good result obtained in the case of hemorrhage, might speak in favor of performing the operation in cases of profuse loss of blood. Here an instantaneous supply of blood will remove the immediate danger, and allow the body to reproduce its own blood by the time the transfused lamb's blood is exhausted. But will a lamb, or any other suitable animal, be at our disposal just when we want it in such cases of emergency? Shall we not find it easier in such cases to get a few ounces of human blood, and to perform the indirect transfusion?—*Medical Examiner.*

